# Rethinking Thoracotomy Analgesia: Paravertebral Block Versus Thoracic Epidural Analgesia in Real-World Practice

**DOI:** 10.7759/cureus.95807

**Published:** 2025-10-31

**Authors:** Muhammad Imran, Jawad Hameed, Obaid U Anwar, Muhammad K Qadeer, Muhammad Sheharyar Ashraf, Abid H Khattak, Fahad R Khan

**Affiliations:** 1 Thoracic Surgery Department, Lady Reading Hospital Medical Teaching Institution, Peshawar, PAK; 2 Anesthesia and Critical Care Department, Lady Reading Hospital Medical Teaching Institution, Peshawar, PAK; 3 Anesthesia Department, Lady Reading Hospital Medical Teaching Institution, Peshawar, PAK; 4 Cardiology Department, Lady Reading Hospital Medical Teaching Institution, Peshawar, PAK

**Keywords:** cough pain, eras, hypotension, open thoracotomy, paravertebral block, thoracic epidural analgesia, urinary retention

## Abstract

Background

Open thoracotomy causes severe dynamic pain that compromises ventilation and cough, with downstream risk of pulmonary complications and delayed recovery. Enhanced Recovery After Surgery (ERAS) pathways emphasize multimodal, opioid-sparing analgesia, but the relative real-world performance of thoracic paravertebral block (PVB) versus thoracic epidural analgesia (TEA) in open thoracotomy remains uncertain and requires appraisal of both effectiveness and safety within ERAS priorities.

Objective

This study aims to compare the analgesic effectiveness and safety of PVB versus TEA after open thoracotomy, with a focus on dynamic pain during the first 48 hours and a composite safety endpoint of hypotension or urinary retention within 72 hours to clarify the clinical benefit-risk profile.

Methods

This retrospective cohort included consecutive adults undergoing open thoracotomy at a tertiary cardiothoracic center between January 1 and December 31, 2024. Both surgical techniques were placed before the induction phase according to program standards: TEA involves a titratable infusion of 0.125% bupivacaine, while PVB consists of a single-shot bupivacaine injection (not repeated), which typically provides six to eight hours of postoperative analgesia. The primary endpoint was time-weighted mean dynamic pain on the numerical rating scale (0-10) over 48 hours. The prespecified safety composite comprised hypotension (≥20% reduction in mean arterial pressure requiring vasopressor therapy) or urinary retention within 72 hours. Secondary outcomes included rest-pain trajectories, opioid consumption (oral morphine equivalents, 0-48 hours), postoperative nausea and vomiting, pulmonary complications, intensive care unit admission within 48 hours, hospital length of stay, and 30-day readmission. Analyses employed propensity-score overlap weighting with robust models and sensitivity checks.

Results

Of 458 screened patients, 380 were analyzed (TEA: 196 (51.6%); PVB: 184 (48.4%)). Pain-score completeness was 97.4% (TEA) and 97.8% (PVB). Dynamic pain declined in both groups (post-anesthesia care unit: TEA 6.8 ± 2.0 vs. PVB 6.2 ± 2.1; 24 h: 5.8 ± 1.9 vs. 5.1 ± 1.8; 48 h: 4.8 ± 1.7 vs. 4.2 ± 1.6). Adjusted mean differences favored PVB at 24 h (-0.6; 95% CI, -0.9 to -0.3) and 48 h (-0.5; 95% CI, -0.8 to -0.2), remaining below the 1-point minimal clinically important difference (consistent with non-inferior dynamic analgesia). The safety composite occurred in 54/196 (27.6%) with TEA versus 26/184 (14.1%) with PVB (adjusted odds ratio 0.44; 95% CI, 0.26-0.72; absolute risk difference -13.5 percentage points; 95% CI, -20.7 to -6.3), with concordant reductions in component outcomes. Opioid use was similar (36 vs. 38 mg; adjusted difference +2 mg; 95% CI, -2 to +6). Other postoperative outcomes were comparable.

Conclusions

In a contemporary ERAS thoracotomy cohort, PVB provided non-inferior dynamic analgesia with superior safety versus TEA, meaningfully reducing hypotension and urinary retention. These findings support PVB as a pragmatic default when hemodynamic stability is prioritized and align with ERAS-aligned benefit-risk considerations.

## Introduction

Open thoracotomy produces meaningful dynamic pain that impairs deep inspiration and effective cough, predisposing patients to postoperative pulmonary complications, such as atelectasis and pneumonia, and slowing recovery [1,2]. Contemporary perioperative pathways for lung surgery therefore emphasize multimodal, opioid-sparing analgesia, with regional techniques as central components, to preserve respiratory mechanics and facilitate early mobilization within Enhanced Recovery After Surgery (ERAS) frameworks [3].

Within this framework, thoracic epidural analgesia (TEA) has long been considered the reference technique, whereas thoracic paravertebral block (PVB) provides a unilateral, segmental somatosensory and sympathetic block that may achieve comparable analgesia with fewer systemic effects. Comparative syntheses, including a Cochrane review and a meta-analysis restricted to randomized trials in thoracotomy, have generally reported similar analgesic efficacy between TEA and PVB, but lower rates of hypotension and urinary retention with PVB [4,5]. However, many prior studies pooled heterogeneous thoracic procedures or minimally invasive approaches, limiting direct applicability to open thoracotomy [4,5]. More recent appraisals situate TEA's benefits against sympathectomy-related risks in ERAS pathways, underscoring the need to balance effectiveness with hemodynamic safety [6].

Because pulmonary toilet is central to convalescence after thoracotomy, movement-evoked ("dynamic") pain is particularly relevant, and contemporary pain-science guidance recommends explicit assessment of dynamic pain rather than relying solely on rest scores [7]. Hemodynamic stability also influences mobilization and organ perfusion, and consensus statements from the Perioperative Quality Initiative have linked perioperative blood-pressure thresholds with adverse outcomes, reinforcing the salience of hypotension as a safety endpoint in analgesic strategy comparisons [8,9]. Urinary retention remains an additional patient-important safety outcome due to its impact on mobility, infection risk, and length of stay.

Against this background, we conducted a pragmatic, real-world comparison of PVB and TEA in adults undergoing open thoracotomy at a high-volume tertiary center, focusing on patient-centered outcomes that align with ERAS priorities. Our primary objective was to compare dynamic pain during the first 48 hours postoperatively; the key safety objective was a composite of hypotension requiring vasopressors or urinary retention within 72 hours. Secondary objectives included rest-pain trajectories, opioid consumption, postoperative nausea and vomiting, pulmonary complications, early intensive care utilization, length of stay, and 30-day readmission. We hypothesized that PVB would provide non-inferior dynamic analgesia with superior safety relative to TEA, clarifying the clinical benefit-risk profile for routine thoracotomy care.

## Materials and methods

Study design and setting

This retrospective observational cohort study was conducted in the Department of Anesthesia at Lady Reading Hospital Medical Teaching Institution (MTI), Peshawar, Pakistan, a high-volume tertiary academic center. Consecutive adults undergoing open thoracotomy between January 1 and December 31, 2024, were included. Reporting adhered to the Strengthening the Reporting of Observational Studies in Epidemiology (STROBE) guidelines to enhance transparency and reproducibility [10] and reflects routine practice within an ERAS-aligned perioperative program.

Participants

Eligible patients were aged ≥18 years, underwent open thoracotomy for lung or pleural surgery, received either TEA or thoracic PVB as the primary regional technique placed before induction per program standards, and had at least one postoperative pain assessment recorded. Exclusion criteria were minimally invasive procedures (video-assisted or robotic), crossover between TEA and PVB during the index anesthetic, chronic preoperative mechanical ventilation, use of additional neuraxial or plexus techniques, or absence of the primary outcome at all time points. Case ascertainment used the perioperative anesthesia information system and thoracic surgery operative log. Data accuracy was cross-checked against anesthesia records, medication charts, and nursing documentation.

Exposures (analgesic techniques)

The exposure was the intraoperative regional technique: TEA (placed before induction and continued postoperatively as a continuous infusion of 0.125% bupivacaine, titrated to effect) or PVB (performed before induction as a single-shot bupivacaine injection, not repeated, typically providing approximately six to eight hours of postoperative analgesia). Both techniques were performed under standard sterile conditions; PVB was placed under ultrasound guidance or with surgeon assistance when appropriate. Protocol elements, including block level, drug concentration/volume, adjuncts, infusion settings, and rescue algorithms, were standardized at the program level and are summarized in Supplement S1 (Protocol Appendix) for reproducibility.

Technique parameters, including block level, local anesthetic type/concentration, adjuncts, infusion settings, and failure/rescue policies, were recorded in the departmental block registry and verified against postoperative orders to capture systemic co-interventions such as multimodal analgesia and wound infiltration. Program-standard practice specified TEA infusion at 0.125% bupivacaine with rate titration for segmental analgesia and hemodynamic tolerance; PVB was single-shot without planned repeat dosing.

Outcomes

The primary endpoint was the time-weighted mean dynamic pain (numerical rating scale (NRS), 0-10) over 48 hours postoperatively, calculated from scores at post-anesthesia care unit (PACU) arrival, 24 hours, and 48 hours using the trapezoidal rule. The safety endpoint was a composite of hypotension or urinary retention within 72 hours. Hypotension was defined as a ≥20% decrease in mean arterial pressure requiring vasopressors, with sensitivity thresholds (MAP <65 mmHg sustained for ≥10-15 minutes) examined for robustness [11]. All patients received an indwelling urinary catheter intraoperatively per institutional protocol, with routine removal on postoperative day one unless clinically indicated. Urinary retention ≤72 hours was defined a priori as bladder ultrasound≥400 mL with inability to void, requiring catheterization or re-catheterization.

Secondary outcomes included postoperative nausea and vomiting (≤48 hours), pulmonary complications through discharge (atelectasis requiring therapy or pneumonia), ICU admission (≤48 hours), hospital length of stay, 30-day readmission, rest-pain trajectories (PACU, 24 hours, 48 hours), and opioid consumption (0-48 hours) standardized to oral morphine equivalents. All definitions and completeness thresholds were prespecified.

Covariates

Baseline and perioperative covariates included demographics, smoking status, body mass index (BMI), chronic obstructive pulmonary disease (COPD)/asthma, diabetes, hypertension, ASA (American Society of Anesthesiologists) class, spirometry (FEV₁ % predicted), procedure type and laterality, duration of one-lung ventilation, intraoperative fluids, and vasopressor use. Covariates were selected for clinical relevance and to align with best practices in propensity-based adjustment [12].

Handling missing data

Missingness was summarized by variable. When required, multiple imputation by chained equations (m=20) was performed, including all exposures, outcomes, and covariates, with passive imputation for derived variables. Diagnostics confirmed convergence, and distributions were compared with complete-case analyses for consistency [13].

Statistical analysis

All analyses were prespecified. Continuous variables are reported as mean ± standard deviation (SD) or median (interquartile range, IQR), and categorical variables as n/N (%). Baseline differences were evaluated using absolute standardized mean differences (SMDs), with values <0.10 indicating adequate balance. Post-weighting covariate balance and propensity overlap were verified before outcome modeling. To minimize confounding by indication, overlap weighting (OW) based on the propensity score was used as the primary adjustment method [14]. The model incorporated prespecified covariates, calendar quarter, and surgeon-team fixed effects, with nonlinear terms modeled using restricted cubic splines. OW emphasizes the region of common support and inherently down-weights patients with extreme propensities (e.g., marked baseline instability), thereby limiting their influence on effect estimates.

For the primary endpoint, adjusted mean differences (aMDs) in dynamic-pain scores were estimated using linear regression. Repeated-measures trajectories were examined with generalized estimating equations (GEE) using robust (sandwich) standard errors and exchangeable versus AR(1) working correlations [15]. For binary outcomes, overlap-weighted logistic regression was applied to generate adjusted odds ratios (aORs) with 95% confidence intervals (CIs), and adjusted risk differences were calculated by marginal standardization. Multiplicity for secondary outcomes was controlled with the Benjamini-Hochberg false discovery rate procedure [16]. No ad hoc post hoc analyses were undertaken; prespecified sensitivity checks address robustness concerns without additional modeling. Prespecified sensitivity analyses included (i) doubly robust estimation (OW plus outcome regression), (ii) calculation of E-values to assess the strength of unmeasured confounding [17], (iii) evaluation of negative-control outcomes [18], (iv) subgroup interaction tests (procedure type, laterality, BMI ≥30, COPD/asthma, TEA continuous infusion vs. PVB single-shot), and (v) per-protocol-like analyses excluding failed blocks or rescues. Two-sided statistical significance was defined as p < 0.05. All analyses were performed in R (version ≥4.3) using validated packages for propensity weighting, balance diagnostics, and GEE.

Sample size and precision

The study was powered to detect a 0.6-point difference in dynamic-pain NRS at 24-48 hours. Assuming SD ≈ 2.0, ~96 patients per group were required for 80% power at α = 0.05 [19]. The achieved cohorts (TEA, n=196; PVB, n=184) exceeded this threshold, providing adequate precision for the planned analyses.

Ethics and transparency

The study was approved by the Institutional Review Board of Lady Reading Hospital, Peshawar, Pakistan (reference 196/LRH/MTI/24). Informed consent was waived due to the retrospective design and use of routinely collected data.

## Results

**Figure 1:**
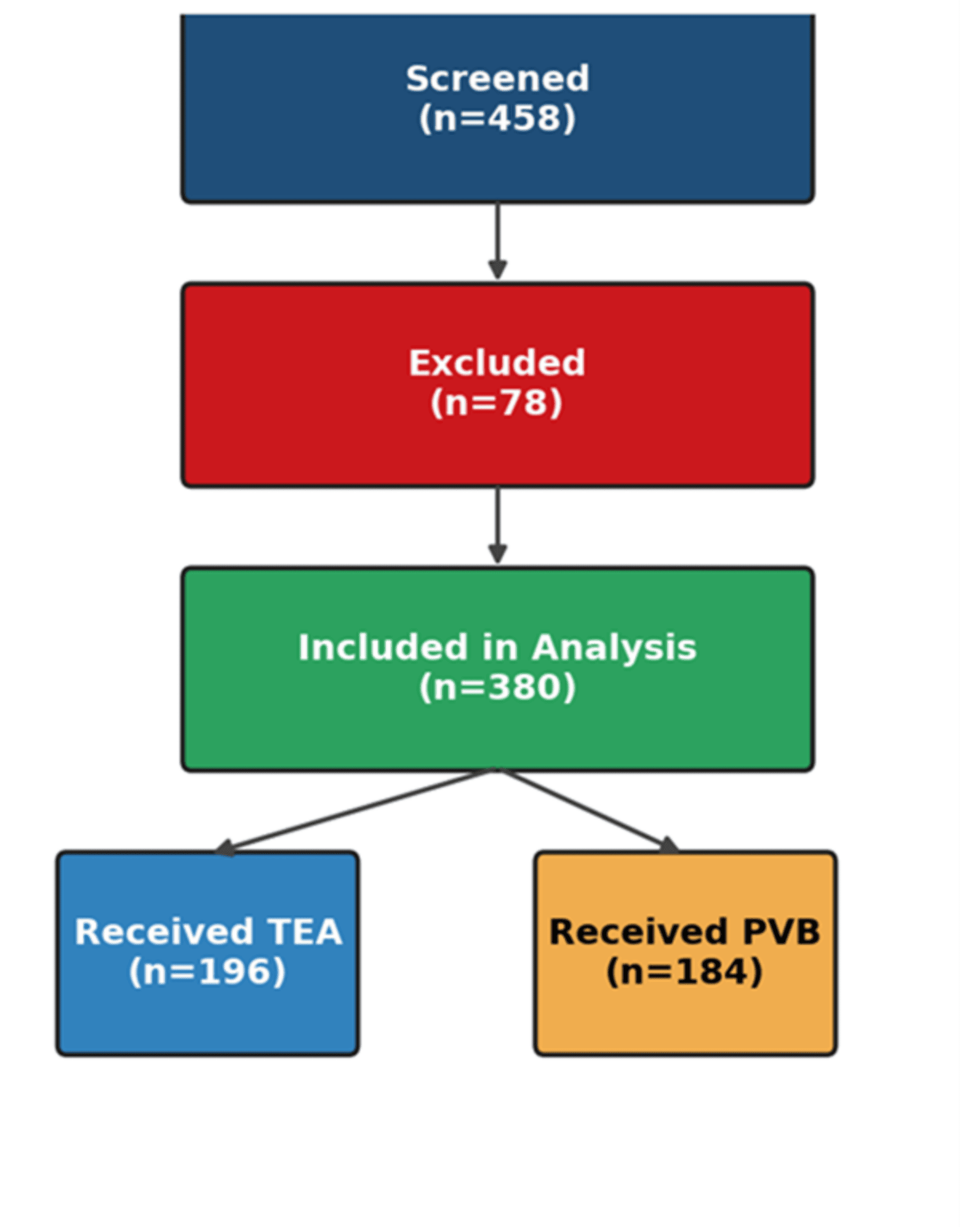
Cohort flow diagram Consecutive adults undergoing open thoracotomy (Jan–Dec 2024) were screened; exclusions (e.g., minimally invasive surgery, crossover before extubation, missing primary outcome) are detailed. Final analytic cohorts received thoracic epidural analgesia (TEA) or paravertebral block (PVB). Percent completeness for pain assessments is shown at each time point.

During the accrual period, 458 thoracotomy cases were screened for eligibility. Of these, 380 patients (83.0%) met the eligibility criteria and were analyzed, including 196 (51.6%) who received TEA and 184 (48.4%) who received thoracic PVB. Exclusions were primarily due to minimally invasive procedures, crossover between techniques before extubation, and absence of the primary outcome. The completeness of the 48-hour pain data was high in both groups (TEA, 191/196 (97.4%); PVB, 180/184 (97.8%)). A cohort flow diagram is provided in Figure 1.

Baseline demographic and clinical characteristics were broadly comparable between the groups, with no clinically meaningful imbalances. The absolute SMDs were generally <0.10, indicating a good balance. Given the observed higher unweighted intraoperative vasopressor use in TEA (29.6% vs. 18.5%), we verified adequate overlap in propensity scores and post-weighting covariate balance before outcome modeling; OW down-weighted extreme propensities, limiting the leverage of hemodynamically unstable outliers. The unweighted characteristics are summarized in Table 1.

**Table 1:** Baseline characteristics (unweighted) *An asterisk indicates statistical significance (P < 0.05). This table summarizes pre-treatment demographics, comorbidities, perioperative variables, and analgesia-related factors for patients receiving thoracic epidural analgesia (TEA) or paravertebral block (PVB) for open thoracotomy. Continuous variables are reported as mean ± SD or median (IQR); categorical variables as n/N (%). Between-group P-values come from the t-test or the Mann-Whitney U (continuous) and the χ² test (categorical), as appropriate. We also report absolute standardized mean differences (SMDs) to indicate balance; SMDs < 0.10 are generally considered well-balanced. Interpretation: use SMDs to judge baseline similarity; P-values are descriptive only at this stage. Notes: Available-case analysis; cell-level denominators (N) are shown if missingness exists. Abbreviations: BMI, body mass index; COPD, chronic obstructive pulmonary disease; ASA, American Society of Anesthesiologists class; others as defined in the Methods section.

Characteristic	TEA (n=196)	PVB (n=184)	P-value
Age, years	59.1 ± 11.8	58.2 ± 12.1	.46
Male sex—n/N (%)	140/196 (71.4)	131/184 (71.2)	.97
BMI, kg/m²	26.7 ± 4.1	26.4 ± 4.3	.47
Current smoker—n/N (%)	64/196 (32.7)	58/184 (31.5)	.80
COPD—n/N (%)	38/196 (19.4)	36/184 (19.6)	.96
Diabetes—n/N (%)	62/196 (31.6)	57/184 (31.0)	.90
Hypertension—n/N (%)	96/196 (49.0)	88/184 (47.8)	.81
ASA class III–IV—n/N (%)	118/196 (60.2)	110/184 (59.8)	.94
FEV₁, % predicted	72 ± 14	73 ± 15	.52
One-lung ventilation, min	126 ± 38	123 ± 36	.33
Intraoperative crystalloids, mL	1600 (1200–2200)	1500 (1100–2100)	.09
Intraoperative vasopressor use—n/N (%)	58/196 (29.6)	34/184 (18.5)	.01*
Resection—lobectomy—n/N (%)	120/196 (61.2)	118/184 (64.1)	.57
Resection—segmentectomy—n/N (%)	40/196 (20.4)	38/184 (20.7)	.93
Resection—pneumonectomy—n/N (%)	22/196 (11.2)	18/184 (9.8)	.64
Resection—decortication—n/N (%)	14/196 (7.1)	10/184 (5.4)	.49
Right-sided thoracotomy—n/N (%)	104/196 (53.1)	95/184 (51.6)	.78

Primary analgesia outcomes

Dynamic pain decreased over time in both patient groups. At the PACU arrival, the mean NRS scores were 6.8 ± 2.0 in the TEA group (196/196, 100.0%) and 6.2 ± 2.1 in the PVB group (184/184, 100.0%). At 24 hours, the mean scores were 5.8 ± 1.9 versus 5.1 ± 1.8, and at 48 hours, 4.8 ± 1.7 in TEA (191/196, 97.4%) compared with 4.2 ± 1.6 in PVB (180/184, 97.8%).

**Figure 2:**
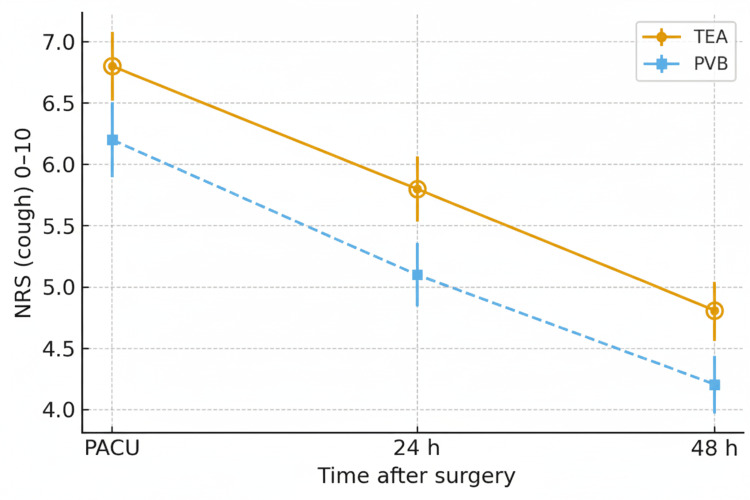
Dynamic-pain trajectories over 48 hours (primary endpoint) Mean numerical rating scale (NRS 0–10) for cough-evoked (dynamic pain) at PACU, 24 h, and 48 h after thoracotomy, by TEA vs. PVB. Lines indicate group means with SD bars. Between-group differences at 24 h and 48 h were estimated using overlap-weighted linear models with robust SEs; negative values favor PVB. Two-sided significance is set at P<0.05.

Adjusted analyses favored PVB at 24 hours (-0.6; 95% CI, -0.9 to -0.3; P < .001) and 48 hours (-0.5; 95% CI, -0.8 to -0.2; P = .002). However, these differences did not exceed the 1-point minimal clinically important difference (MCID) and thus are consistent with non-inferior dynamic analgesia. Dynamic-pain trajectories are displayed in Figure 2, and the analgesia plus opioid use is summarized in Table 2.

**Table 2:** Analgesia and opioid outcomes (primary domain) This table presents pain intensity (NRS 0–10) at PACU, 24 h, and 48 h—for dynamic and rest pain—plus cumulative opioid consumption (0–48 h) in OME (mg) for TEA vs. PVB. Alongside crude summaries, the table reports adjusted between-group differences estimated with propensity-score overlap–weighted linear models and heteroskedasticity-robust standard errors, yielding adjusted mean differences (aMD) with 95% CIs. Directionality: negative aMD values indicate lower pain/OME with PVB. Two-sided P < 0.05 was considered statistically significant; when multiple secondary pain endpoints are shown, FDR control was applied as indicated. NRS anchors: 0 = no pain, 10 = worst imaginable. OME conversions followed standard equi-analgesic ratios. Per-time-point denominators (n/N) are shown.

Metric	TEA	PVB	Adjusted difference	95% CI	P-value
NRS (dynamic), PACU—mean ± SD (n/N, %)	6.8 ± 2.0 (196/196, 100.0)	6.2 ± 2.1 (184/184, 100.0)	–0.5	–0.8 to –0.2	.003*
NRS (dynamic), 24 h—mean ± SD (n/N, %)	5.8 ± 1.9 (196/196, 100.0)	5.1 ± 1.8 (184/184, 100.0)	–0.6	–0.9 to –0.3	< .0001*
NRS (dynamic), 48 h—mean ± SD (n/N, %)	4.8 ± 1.7 (191/196, 97.4)	4.2 ± 1.6 (180/184, 97.8)	–0.5	–0.8 to –0.2	.002*
NRS (rest), PACU—mean ± SD (n/N, %)	4.8 ± 1.9 (196/196, 100.0)	4.5 ± 1.8 (184/184, 100.0)	–0.2	–0.5 to 0.1	.18
NRS (rest), 24 h—mean ± SD (n/N, %)	3.7 ± 1.6 (196/196, 100.0)	3.2 ± 1.5 (184/184, 100.0)	–0.4	–0.7 to –0.1	.01*
NRS (rest), 48 h—mean ± SD (n/N, %)	3.0 ± 1.5 (191/196, 97.4)	2.7 ± 1.3 (180/184, 97.8)	–0.3	–0.5 to –0.1	.02*
Opioids 0–48 h, OME mg—median (IQR) (n/N, %)	36 (24–54) (196/196, 100.0)	38 (26–57) (184/184, 100.0)	+2	–2 to +6	.32

Complementary analgesic outcomes

**Figure 3:**
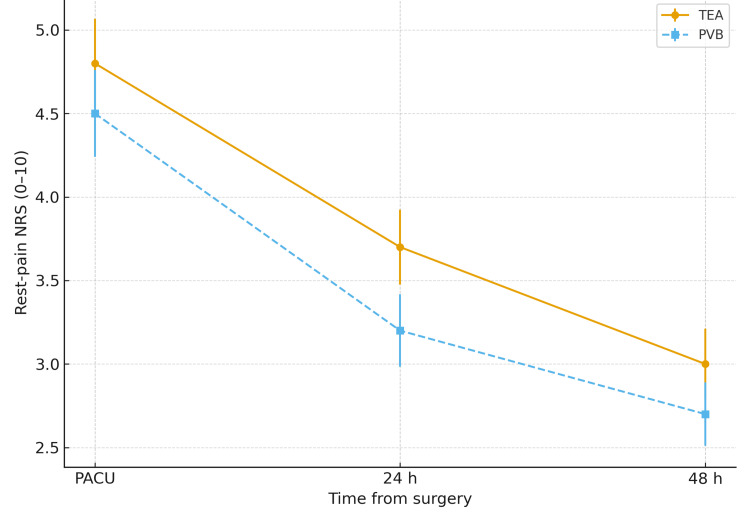
Rest-pain trajectories over 48 hours (complementary analgesia panel) Mean rest NRS (0–10) at PACU, 24 h, and 48 h, by TEA vs. PVB. Analysis mirrors Figure 2. These secondary outcomes were FDR-adjusted (Benjamini–Hochberg); negative adjusted differences favor PVB.

Rest-pain trajectories are shown in Figure 3. At PACU arrival, mean rest-pain scores were 4.8 ± 1.9 in the TEA group and 4.5 ± 1.8 in the PVB group. At 24 hours, mean scores were 3.7 ± 1.6 versus 3.2 ± 1.5, and at 48 hours, 3.0 ± 1.5 (TEA, n = 191) compared with 2.7 ± 1.3 (PVB, n = 180). Overlap-weighted models favored PVB at 24 hours (aMD -0.4; 95% CI, -0.7 to -0.1; P = .01) and 48 hours (-0.3; 95% CI, -0.5 to -0.1; P = .02), with no difference at PACU (-0.2; 95% CI, -0.5 to 0.1; P = .18). These differences were small and remained below the 1-point MCID, aligning with the primary finding of non-inferior dynamic analgesia.

Opioid consumption over the first 48 hours, standardized to oral morphine equivalents (OME), was not graphed but is summarized in Table 2. Median (IQR) use was 36 (24-54) mg in the TEA group and 38 (26-57) mg in the PVB group, with an adjusted difference of +2 mg (95% CI, -2 to +6; P = .32), indicating no material between-group difference in rescue opioid requirements.

Safety and other postoperative outcomes

**Figure 4:**
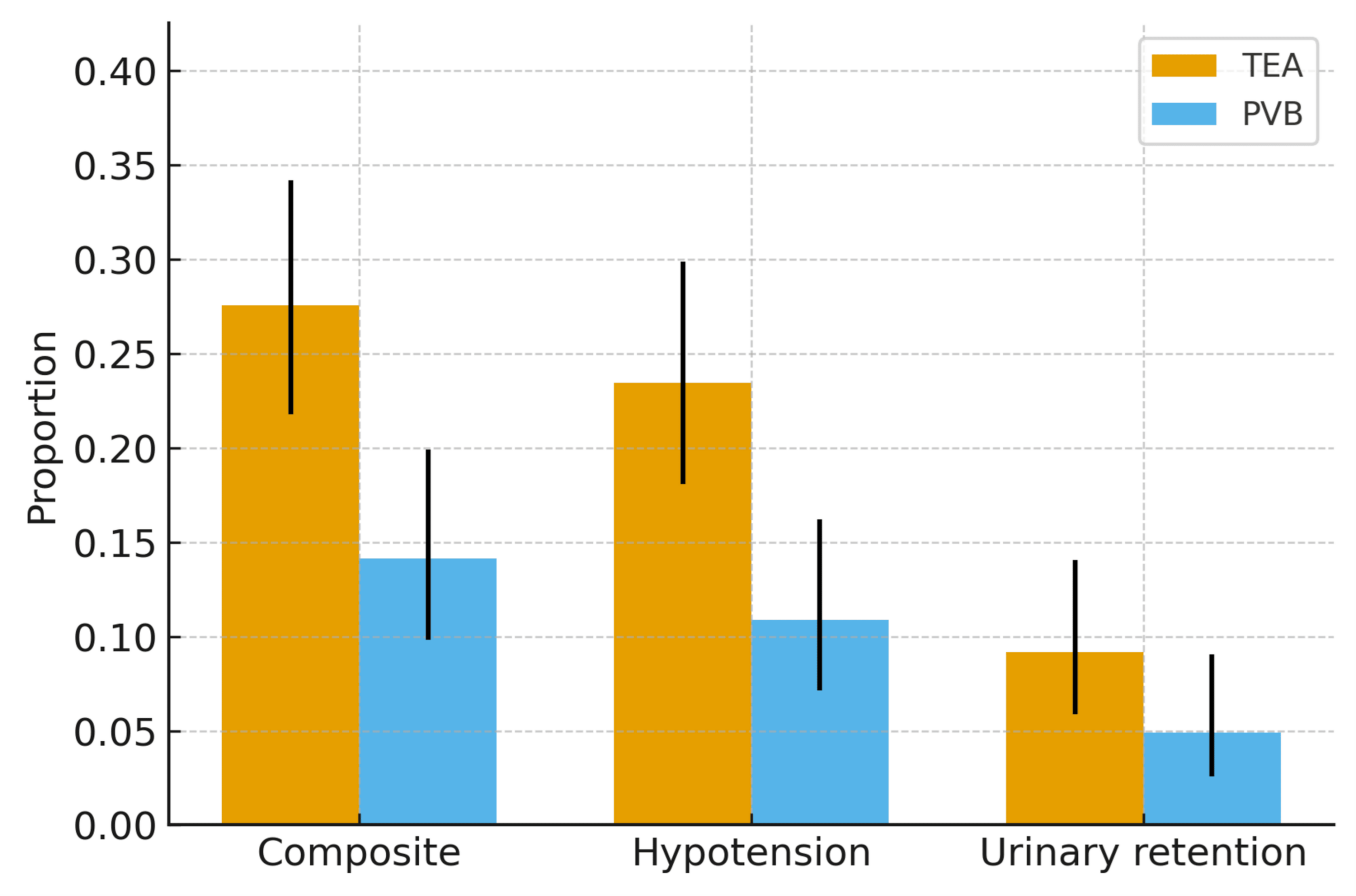
Safety composite and components (72 hours) Grouped bars display overlap-weighted event proportions for the composite safety endpoint—hypotension requiring vasopressor support or urinary retention—and for each component separately. Orange = TEA; blue = PVB. Black error bars indicate 95% confidence intervals for the overlap-weighted estimates. Adjusted between-group effects were obtained using propensity-score overlap–weighted logistic regression with heteroskedasticity-robust standard errors (covariates per Methods) and are reported as adjusted odds ratios (aOR) with 95% CIs in the Results; aOR < 1.00 favors PVB. Two-sided P<0.05 defined statistical significance; no multiplicity adjustment was applied to safety endpoints.

Within 72 hours, the safety composite of hypotension requiring vasopressor therapy or urinary retention occurred in 54/196 (27.6%) patients in the TEA group versus 26/184 (14.1%) in the PVB group. This favored PVB (adjusted odds ratio (aOR) 0.44; 95% CI, 0.26-0.72; P = .001), corresponding to an adjusted absolute risk difference of -13.5 percentage points (95% CI, -20.7 to -6.3). Unadjusted and overlap-weighted proportions are displayed in Figure 4.

The component outcomes were directionally consistent: hypotension occurred in 23.5% of TEA versus 10.9% of PVB patients (aOR 0.41; 95% CI, 0.23-0.72; P = .002), and urinary retention in 9.2% versus 4.9% (aOR 0.50; 95% CI, 0.22-1.11; P = .09). The concordant direction of both components with the composite supports a superior safety profile for PVB. Adjusted effects for all endpoints are summarized in Table 3.

**Table 3:** Safety and postoperative outcomes * Statistical significance is defined as P < .05. Values are expressed as n/N (%) or median (IQR) unless otherwise indicated. Adjusted effects were derived from overlap-weighted regression models with robust standard errors. Logistic regression was used for binary outcomes and linear regression for continuous outcomes. Hypotension was defined as a ≥20% reduction in mean arterial pressure requiring vasopressor therapy; a robustness check using MAP < 65 mmHg sustained ≥10–15 minutes yielded consistent inferences (data not shown). Adjusted absolute risk differences for statistically significant outcomes are reported in the main text. aOR = adjusted odds ratio; Δ = adjusted mean difference; LOS = length of stay; PONV = postoperative nausea and vomiting.

Outcome	TEA	PVB	Adjusted Effect (Model)	95% CI	P-value
Safety composite (≤72 h)	54/196 (27.6)	26/184 (14.1)	aOR 0.44 (logistic)	0.26–0.72	.001
Hypotension (≤72 h)	46/196 (23.5)	20/184 (10.9)	aOR 0.41 (logistic)	0.23–0.72	.002
Urinary retention (≤72 h)	18/196 (9.2)	9/184 (4.9)	aOR 0.50 (logistic)	0.22–1.11	.09
PONV (≤48 h)	26/196 (13.3)	23/184 (12.5)	aOR 0.94 (logistic)	0.52–1.70	.84
Pulmonary complications (through discharge)	22/196 (11.2)	16/184 (8.7)	aOR 0.76 (logistic)	0.39–1.47	.42
ICU admission (≤48 h)	18/196 (9.2)	12/184 (6.5)	aOR 0.69 (logistic)	0.32–1.45	.33
Hospital LOS, days—median (IQR)	5 (4–7)	5 (4–6)	Δ –0.3 (linear)	–0.6 to 0.0	.06
30-day readmission	12/196 (6.1)	10/184 (5.4)	aOR 0.87 (logistic)	0.37–2.05	.77

Other outcomes were comparable between groups, including postoperative nausea and vomiting (≤48 h: 13.3% vs. 12.5%; aOR 0.94; 95% CI, 0.52-1.70; P = .84), pulmonary complications (11.2% vs. 8.7%; aOR 0.76; 95% CI, 0.39-1.47; P = .42), intensive care unit admission within 48 hours (9.2% vs. 6.5%; aOR 0.69; 95% CI, 0.32-1.45; P = .33), hospital length of stay (median (IQR) 5 (4-7) vs. 5 (4-6) days; adjusted difference -0.3 days; 95% CI, -0.6 to 0.0; P = .06), and 30-day readmission (6.1% vs. 5.4%; aOR 0.87; 95% CI, 0.37-2.05; P = .77). No ad hoc post hoc analyses were undertaken; pre-specified checks yielded consistent inferences.

Technique-specific adverse events

Serious technique-related complications were rare. No cases of epidural hematoma (0/380, 0.0%) or local anesthetic systemic toxicity (0/380, 0.0%) were observed. A small pneumothorax occurred after PVB in 1/184 patients (0.5%) and was managed conservatively. Catheter failure requiring a change of technique occurred in 4/196 patients (2.0%) in the TEA group and 6/184 patients (3.3%) in the PVB group.

Overall, these complementary findings reinforce that PVB provides non-inferior analgesia with a superior safety profile compared with TEA, aligning with ERAS-aligned benefit-risk priorities.

## Discussion

In this contemporary ERAS thoracotomy cohort, PVB achieved dynamic-pain trajectories and opioid use comparable to TEA while meaningfully reducing a composite of hypotension or urinary retention. This pattern, non-inferior dynamic analgesia with superior safety, aligns with high-quality comparative evidence in thoracic surgery and extends its relevance to open thoracotomy within a real-world program. Meta-analytic data indicate that alternatives to TEA, including PVB and intercostal techniques, achieve noninferior early pain control, consistent with the modest, sub-MCID differences observed here at 24-48 hours (aMD ≤0.6 NRS points) [20]. A recent randomized trial in thoracoscopic surgery directly comparing PVB with TEA also reported overlapping pain outcomes, reinforcing that dense neuraxial blockade is not uniquely required for effective analgesia when multimodal care is standardized [21]. These findings are notable given our explicitly standardized techniques: TEA was placed before induction and continued as a titratable 0.125% bupivacaine infusion throughout the early postoperative period, whereas PVB was performed before induction as a single-shot bupivacaine injection (not repeated) with an expected six- to eight-hour duration, yet dynamic pain remained comparable on a time-weighted basis across 0-48 hours. Consistent with our analytic strategy, OW targeted the region of common support and down-weighted extreme propensities, supporting that estimates were not driven by hemodynamically unstable outliers; the concordant directions of both safety components reinforce this interpretation.

Safety findings in this study mirror comparative syntheses in lung surgery: PVB is associated with lower rates of hypotension and urinary retention without compromising analgesia [22]. Procedure-specific guidance has evolved accordingly. The PROSPECT recommendations for VATS endorse paravertebral and paraspinal plane blocks as first-line options and no longer favor routine TEA, explicitly weighing adverse-effect profiles, guidance that resonates with our observed hemodynamic advantage while maintaining dynamic-pain control [23]. Implementation studies further demonstrate that PVB can be delivered reliably by anesthesiologists under ultrasound guidance or by surgeons under thoracoscopic vision, with noninferior outcomes, highlighting operational flexibility for ERAS pathways [24]. Clinically, the direction and magnitude of benefit in our cohort (adjusted odds ratio for the safety composite: 0.44; absolute risk difference: −13.5 percentage points) align with ERAS priorities that emphasize early mobilization, hemodynamic stability, and avoidance of catheter-related morbidity.

Comparative networks that include PVB, intercostal nerve blocks, serratus/pectoralis, and erector spinae plane blocks consistently rank TEA among the least favorable strategies for tolerability while showing similar analgesic outcomes across techniques [25]. Educational reviews specific to open thoracotomy increasingly depict PVB as a preferred approach when hemodynamic stability and ward feasibility are priorities, citing equivalent dynamic-pain relief, fewer adverse effects, and simpler monitoring, practical advantages that align with our findings [26]. Although serious neuraxial complications are rare, large database analyses indicate higher rates in non-obstetric surgical populations; while none occurred here, recognition of this risk underscores the value of peripherally based alternatives [27]. Taken together, our data support selecting PVB as a pragmatic default when maintaining arterial pressure and minimizing urinary retention are prioritized, with no apparent trade-off in dynamic pain or opioid burden. This benefit-risk profile is particularly pertinent for ERAS programs seeking reliable ward-based pathways and early ambulation.

Recent viewpoints in thoracic anesthesia describe a gradual shift toward targeted paraspinal techniques, supported by accumulating randomized and synthetic evidence as well as operational benefits such as unilateral coverage, broader eligibility, and compatibility with anticoagulation pathways [28,29]. Against that backdrop, our results contribute thoracotomy-specific evidence: PVB maintained functional analgesia while halving the incidence of early adverse events most plausibly linked to sympathetic blockade. The congruence of our real-world, overlap-weighted estimates with prior randomized syntheses strengthens external validity and suggests that standardized multimodal co-analgesia can offset the finite duration of single-shot PVB without necessitating routine neuraxial catheterization.

Limitations

Several limitations temper inference. The retrospective, single-center design permits residual confounding despite prespecified propensity-score OW, balance diagnostics, and sensitivity analyses. OW targets the region of common support and down-weights extreme propensities (e.g., hemodynamically unstable outliers), but unmeasured factors influencing the choice of TEA versus PVB (such as clinician preference or perceived intraoperative risk) could still bias estimates. Technique heterogeneity (single-shot vs. catheterized PVB; variable TEA dosing and adjuncts) reflects real-world practice and may attenuate or amplify observed differences. Although techniques were standardized at the program level (pre-induction TEA with a continuous 0.125% bupivacaine infusion; pre-induction single-shot PVB without repeat dosing), case-level documentation of exact infusion rates and adjuncts was variably detailed; consequently, we reported program standards and did not attempt dose-response modeling. Similarly, co-analgesic and rescue pathways were protocolized, yet patient-level adherence inevitably varied, potentially introducing nondifferential noise.

Pain scores, although systematically collected, remain patient-reported; missingness was low but present. Our primary endpoint (time-weighted mean dynamic pain using scheduled PACU, 24-hour, and 48-hour assessments) was chosen to maximize completeness in routine care; intermediate ward scores were inconsistently charted and therefore not analyzed. While the time-weighted approach captures early functional pain, it may underrepresent short-lived peaks between scheduled assessments. Safety endpoints were abstracted from electronic records and may under-capture transient hypotension, yet the concordant reductions in both hypotension and urinary retention strengthen internal validity. Definitions of urinary retention and catheter policy were prespecified and applied uniformly, but ascertainment bias remains a possibility in borderline cases. Event counts for some secondary outcomes (e.g., urinary retention as a component endpoint) limited precision, so the absence of statistical significance should not be overinterpreted as equivalence.

## Conclusions

PVB provided non-inferior analgesia to TEA for dynamic pain over 48 hours after open thoracotomy while meaningfully reducing hypotension and urinary retention, indicating a superior safety profile. In a program using pre-induction TEA with a continuous 0.125% bupivacaine infusion versus pre-induction single-shot PVB, sub-MCID differences in dynamic pain, alongside a clinically important safety advantage, support PVB as a pragmatic default within ERAS pathways when hemodynamic stability and ward feasibility are prioritized. Future studies should prioritize thoracotomy-specific randomized comparisons of catheterized PVB versus standardized TEA, include uniform technique reporting (e.g., urinary retention definitions/catheter policy and hemodynamic endpoints), and emphasize patient-important outcomes, including pulmonary complications, quality of recovery, return to function, and chronic pain, to refine patient- and procedure-level selection.
